# Radiotherapy for Adrenal Metastasis from Hepatocellular Carcinoma: A Multi-Institutional Retrospective Study (KROG 13-05)

**DOI:** 10.1371/journal.pone.0152642

**Published:** 2016-03-29

**Authors:** Jinhong Jung, Sang Min Yoon, Hee Chul Park, Taek-Keun Nam, Jinsil Seong, Eui Kyu Chie, Tae Hyun Kim, Mi-Sook Kim, Chul Yong Kim, Hong Seok Jang, Jong Hoon Kim

**Affiliations:** 1 Department of Radiation Oncology, Kyung Hee University Medical Center, Kyung Hee University School of Medicine, Seoul, Republic of Korea; 2 Department of Radiation Oncology, Asan Liver Center, Asan Medical Center, University of Ulsan College of Medicine, Seoul, Republic of Korea; 3 Department of Radiation Oncology, Samsung Medical Center, Sungkyunkwan University School of Medicine, Seoul, Republic of Korea; 4 Department of Medical Device Management and Research, SAIHST, Sungkyunkwan University, Seoul, Republic of Korea; 5 Department of Radiation Oncology, Chonnam National University Hospital, Gwang-Ju, Republic of Korea; 6 Department of Radiation Oncology, Yonsei University College of Medicine, Seoul, Republic of Korea; 7 Department of Radiation Oncology, Seoul National University College of Medicine, Seoul, Republic of Korea; 8 Research Institute and Hospital, National Cancer Center, Goyang, Republic of Korea; 9 Department of Radiation Oncology, Korea Institute of Radiological and Medical Sciences, Seoul, Republic of Korea; 10 Department of Radiation Oncology, Korea University College of Medicine, Seoul, Republic of Korea; 11 Department of Radiation Oncology, The Catholic University of Korea College of Medicine, Seoul, Republic of Korea; School of Medicine, Fu Jen Catholic University, TAIWAN

## Abstract

Although the adrenal glands are not common sites of metastasis from hepatocellular carcinoma (HCC), this metastasis can be met in patients with advanced HCC in some clinical settings. However, the effectiveness of radiotherapy against such metastases is unclear. Therefore, we performed the present multi-institutional study to investigate tumor response, overall survival (OS), treatment-related toxicity, and prognostic factors after radiotherapy. We retrospectively reviewed 134 patients who completed a planned radiotherapy for their adrenal metastases. Complete response was noted in 6 (4.3%), partial response in 48 (34.0%), and stable disease in 78 patients (55.3%). The median OS was 12.8 months, and the 1-, 2-, and 5-year OS rates were 53.1%, 23.9%, and 9.3%, respectively. Grade 3 anorexia occurred in 2 patients, grade 3 diarrhea in 1, and grade 3 fatigue in 1. Multivariate analyses revealed that the following factors had significant effects on OS: controlled intrahepatic tumor; controlled extrahepatic metastasis; and Child-Pugh class A. Although patients with adrenal metastasis from HCC had poor OS, radiotherapy provided an objective response rate of 38.3% and disease stability of 93.6%, with minimal adverse events. Therefore, radiotherapy for these patients could represent a good treatment modality, especially for patients with controlled intrahepatic tumors, controlled extrahepatic metastasis, and good hepatic function.

## Introduction

With advances in diagnosis and treatment of hepatocellular carcinoma (HCC), survival outcomes have increased considerably over time. However, most patients suffer from frequent tumor recurrences as well as chronic liver disease, and thus the prognosis of this disease is still poor. Accordingly, HCC is still the second most common cause of death from cancer according to a recent worldwide statistical report [1]. It has generally been thought that extrahepatic metastasis is not uncommon and is observed more frequently due to improved diagnostic methods and prolonged patient survival [2]. The most frequent metastatic site is the lung, while other common metastatic sites include lymph nodes, bone, and adrenal glands [2–4].

As mentioned above, although the adrenal glands are not common metastatic sites of HCC, with an incidence of 8% in autopsies and 8.8–16.9% in HCC patients with extrahepatic metastasis, this metastasis can be met in patients with advanced HCC in some clinical settings [2,4–9]. Various treatment modalities have been performed for adrenal metastasis; surgical resection provided a survival rate of 51.3% at 1 year and a median survival of 21.4 months, while non-surgical treatments, including transarterial chemoembolization (TACE), percutaneous ethanol injection (PEI), radiotherapy, and systemic chemotherapy, provided a survival rate of 42–42.5% at 1 year and a median survival of 11.1–13.6 months [10–13]. Nevertheless, an optimal treatment strategy has yet to be established.

It is also important to provide relief from symptoms to these patients as well as to prolong their overall survival (OS). Although there have been only a few studies on radiotherapy for adrenal metastasis of HCC, these have demonstrated effective palliation, especially pain relief [11,13]. However, the effectiveness of radiotherapy remains unclear from these studies with respect to tumor response, survival benefit, or subgroup(s) likely to derive benefit, because the sample sizes were relatively small. Considering the relatively rarity of the clinical situation of radiotherapy for adrenal metastasis of HCC, it was impossible to determine the effects of radiotherapy through a prospective randomized study. Given this state of affairs, we performed this retrospective multi-institutional study with subjects obtained from 9 hospitals of the Korean Radiation Oncology Group (KROG). The purpose of this study was to investigate tumor response, OS, and treatment-related toxicity, and to find prognostic factors for OS after radiotherapy.

## Materials and Methods

This retrospective study was performed using the medical records of radiotherapy for adrenal metastasis from HCC between May 2000 and December 2012 in 9 hospitals of the KROG. The inclusion criteria for this study were as follows: (1) HCC was diagnosed based on the guidelines of the American Association for the Study of Liver Diseases; (2) adrenal metastasis was diagnosed clinically by computed tomography (CT), and/or magnetic resonance imaging (MRI), and/or angiograph; (3) age ≥ 20 years; (4) completion of planned radiotherapy; (5) treatment with modern radiotherapy techniques, including three-dimensional conformal radiotherapy (3D-CRT) or intensity-modulated radiotherapy or stereotactic body radiotherapy (SBRT); (6) no prior history of treatment for adrenal metastasis. The diagnostic criteria for adrenal metastasis were as follows: (1) an enhancing soft tissue mass in the adrenal gland with/without central necrosis; (2) a serial changes in size of adrenal mass on CT or MRI; or (3) the changes in tumor marker levels such as alpha-fetoprotein. The present study was approved by the KROG and the Institutional Review Boards of each participant hospital, and written informed consent was waived because of the retrospective nature of the study.

Tumor response was defined at the time of each maximal response according to the Response Evaluation Criteria in Solid Tumors criteria (RECIST version 1.1) using CT and/or MRI. A complete response (CR) was defined as disappearance of all target lesions. A partial response (PR) required at least a 30% decrease in the sum of diameters of target lesions, taking as reference the baseline sum diameters. Progressive disease (PD) was defined as at least a 20% increase in the sum of diameters of target lesions, taking as reference the smallest sum on study. Stable disease (SD) indicated neither sufficient shrinkage to qualify for PR nor sufficient increase to qualify for PD, taking as reference the smallest sum of diameters. Objective response was defined as CR or PR. Controlled intrahepatic tumor was defined as no evidence of tumor at the time of diagnosis of adrenal metastasis. Controlled extrahepatic metastasis was defined as a solitary adrenal metastasis or no evidence of viable extrahepatic metastasis except adrenal metastasis with previous treatments. OS was estimated from the date of the start of radiotherapy to the date of death or the last follow-up examination. The existence of a variable-effect relationship was confirmed by logistic regression analysis. The probability of cumulative survival was calculated using the Kaplan-Meier method and was compared using the log-rank test. Multivariate analysis was performed with a Cox proportional hazards model. A *p*-value < 0.05 was considered statistical significant. Statistical analyses were performed using the Statistical Package for Social Science, version 18.0 (SPSS Inc., Chicago, IL, USA). Adverse effects related to radiotherapy were graded according to the Common Terminology Criteria for Adverse Events (CTCAE version 4.2).

## Results

### Patient characteristics

A total of 152 patients underwent radiotherapy for adrenal metastasis from HCC during the study period. Among them, 18 patients were excluded due to the following reasons: 8 patients who had no medical record immediately after radiotherapy; 4 patients who did not complete planned radiotherapy; 4 patients who received other treatment for adrenal metastasis (3 received surgery and 1 received TACE) before radiotherapy; and 2 patients who underwent 2-dimensional radiotherapy technique. Therefore, a total of 134 patients (lesion numbers: 142) with adrenal metastases from HCC were included in the present study (Table 1). The study population was mostly male (92.5%), with a median age of 59 years (range: 35–76 years). Most patients had an Eastern Cooperative Oncology Group performance status of 0 (38.8%) or 1 (46.3%) and were chronic carriers of the hepatitis B virus (87.3%). One hundred and fourteen (85.1%) patients had liver function of Child-Pugh class A. Thirty (21.7%) patients had no evidence of intrahepatic HCC, and 93 (67.4%) patients showed no evidence of extrahepatic metastasis except to adrenal glands at the time of diagnosis of adrenal metastasis. The median period from initial diagnosis of HCC to diagnosis of adrenal metastasis was 12.5 months (range: 0–106.2 months). Among 142 lesions, 86 (60.6%) were located at the right adrenal gland, including 8 bilateral adrenal gland metastases. The median tumor size was 4.7 cm (range: 1.0–15.0 cm). The median radiation dose was 45 Gy (range: 20–66 Gy) with median fraction size of 2.5 Gy (range: 1.8–15 Gy). Among 125 patients who received conventional fractionated 3D-CRT, the median radiation dose was 45 Gy (range: 20–66 Gy) with median fraction size of 2.5 Gy (range: 1.8–5 Gy). Because there was a diversity of total dose and fraction size, we used the biologically effective dose (BED) for analysis, and found that the median BED was 58.5 Gy_10_ (range: 25–112.5 Gy_10_). Among 134 patients, 15 (10.5%) patients received systemic treatments (12 sorafenib, 1 sunitinib, 1 fluorouracil, and 1 fluorouracil and oxaliplatin).

**Table 1:** Patient characteristics and summary of treatment.

Variables		No (%)
Gender^a^	Male	124 (92.5)
	Female	10 (7.5)
Age (years)^a^	Median (range)	59 (35–76)
ECOG performance status^a^	0–1	114 (85.1)
	2–3	20 (14.9)
Viral etiology^a^	Hepatitis B virus	117 (87.3)
	Hepatitis C virus	9 (6.7)
	NBNC	9 (6.7)
Intrahepatic control^b^	NED	30 (21.7)
	Viable, but SD	64 (46.4)
	Uncontrolled or not available	44 (31.9)
Extrahepatic control^b^	NED	93 (67.4)
	Viable, but SD	23 (16.7)
	Uncontrolled	22 (15.9)
Time to adrenal metastasis^b^	Median (range)	12.5 (0–106.2)
Tumor size (cm)^c^	Median (range)	4.7 (1.0–15.0)
Alpha-fetoprotein (ng/mL)^b^	Median (range)	155.0 (1.1–327000)
Child-Pugh class^a^	A	114 (85.1)
	B	19 (14.2)
	C	1 (0.7)
Total dose (Gy)^c^	Median (range)	45 (20–66)
Fraction size (Gy)^c^	Median (range)	2.5 (1.8–15)
BED (Gy_10_)^c^	Median (range)	58.5 (25–112.5)
Radiotherapy technique^c^	3D-CRT	125 (88.0)
	SBRT	9 (6.3)
	IMRT	8 (5.6)

ECOG = Eastern Cooperative Oncology Group; NBNC = non B non C; NED = no evidence of disease; SD = stable disease; TACE = transarterial chemoembolization; RFA = radiofrequency ablation; PEI = percutaneous ethanol injection; BED = biologically effective dose; 3D-CRT = three-dimensional conformal radiotherapy; SBRT = stereotactic body radiotherapy; IMRT = intensity-modulated radiotherapy.

^a^One hundred and thirty four patients were analyzed.

^b^One hundred and thirty eight cases were analyzed because 4 patients received RT at different time each other.

^c^One hundred and forty two lesions were analyzed.

### Tumor response and survival outcomes

CT or MRI at maximal response was evaluated for 141 lesions. CR was noted in 6 (4.3%), and PR in 48 (34.0%), yielding an objective response rate of 38.3% according to the RECIST criteria (Table 2). Except for 9 patients who suffered disease progression, most patients achieved local tumor control without progression after radiotherapy. There was no significant correlation between response rate and radiation dose (BED: *p* = 0.199). The median time from radiotherapy to maximal response for patients who achieved objective response was 3 months (range: 1–8 months).

**Table 2:** Response rate after radiotherapy for adrenal metastasis from HCC.

Response (n = 141)	No. of lesions (%)
Complete response	6 (4.3)
Partial response	48 (34.0)
Stable disease	78 (55.3)
Progressive disease	9 (6.4)
Objective response rate	54 (38.3)

There was no significant correlation between response rate and radiation dose (BED: *p* = 0.199) in the logistic regression analysis.

**Fig 1:**
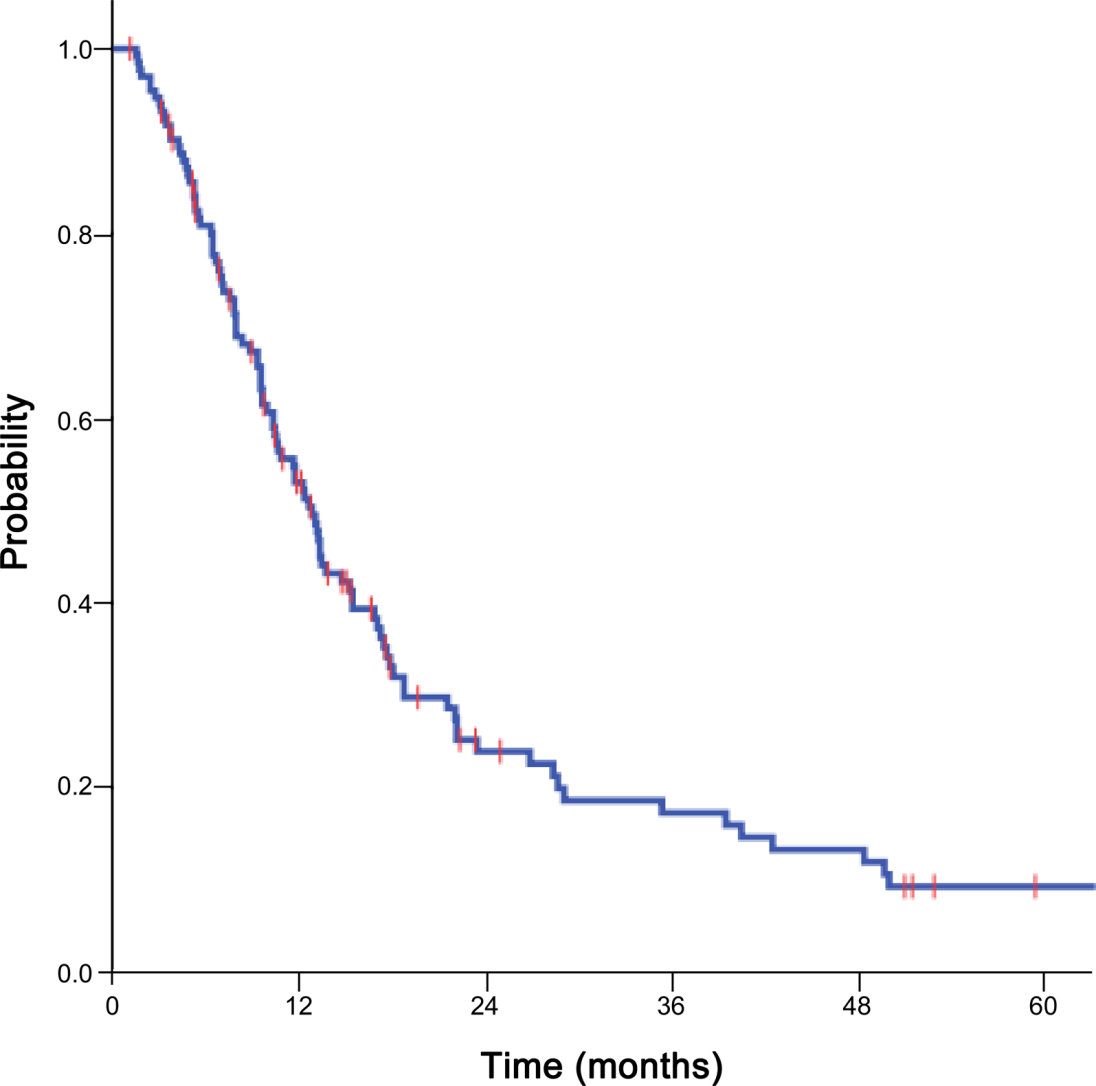
Overall survival curve. Overall survival curve for patients with adrenal metastasis from hepatocellular carcinoma receiving radiotherapy.

The median follow-up period for all patients was 10.7 months (range: 1.1–104.2 months) and that for survivors was 13.8 months (range: 1.1–104.2 months). At the time of analysis, 33 patients were alive and 101 patients were deceased. The median OS was 12.8 months, and the 1, 2, and 5 year OS rates were 53.1%, 23.9%, and 9.3%, respectively (Fig 1).

Nine patients underwent SBRT with median BED of 60 Gy_10_ (range: 42.6–112.5 Gy_10_) and median fraction size of 10 Gy (range: 5.5–15.5 Gy), among them, 3 patients experienced adrenal metastasis progression at 1.3 months, 5.7 months, and 38.4 months after radiotherapy, respectively.

### Independent prognostic factors for overall survival

The univariate and multivariate analyses are summarized in Table 3. By univariate analysis, controlled intrahepatic tumor, controlled extrahepatic metastasis except adrenal metastasis, tumor size less than 4.7 cm (median tumor size), alpha-fetoprotein value less than 400 ng/mL, and Child-Pugh class A were related to favorable OS. Multivariate analyses revealed that the following factors had significant effects on OS: controlled intrahepatic tumor (hazard ratio [HR] = 0.427; 95% CI, 0.250–0.728; *p* = 0.002); controlled extrahepatic metastasis (HR = 0.568; 95% CI, 0.368–0.877; *p* = 0.011); and Child-Pugh class A (HR = 0.463; 95% CI, 0.271–0.792; *p* = 0.005).

**Table 3:** Univariate and multivariate analysis of prognostic factors associated with overall survival.

Variables		Univariate	Multivariate
Gender	Male vs. Female	0.193	.
Age (years)	<60 vs. ≥60	0.961	.
ECOG performance status	0–1 vs. 2–3	0.382	.
Intrahepatic control	NED vs. SD or Uncontrolled	0.003	0.002
Extrahepatic control	NED vs. SD or Uncontrolled	0.005	0.011
Time to adrenal metastasis	<1 year vs. ≥1 year	0.461	.
Tumor size (cm)	<4.7 vs. ≥4.7	0.020	.
Tumor location	Right vs. Left	0.915	.
Alpha-fetoprotein (ng/mL)	<400 vs. ≥400	0.031	0.065
Child-Pugh class	A vs. B,C	0.002	0.005

ECOG = Eastern Cooperative Oncology Group; NED = no evidence of disease; SD = stable disease.

**Fig 2:**
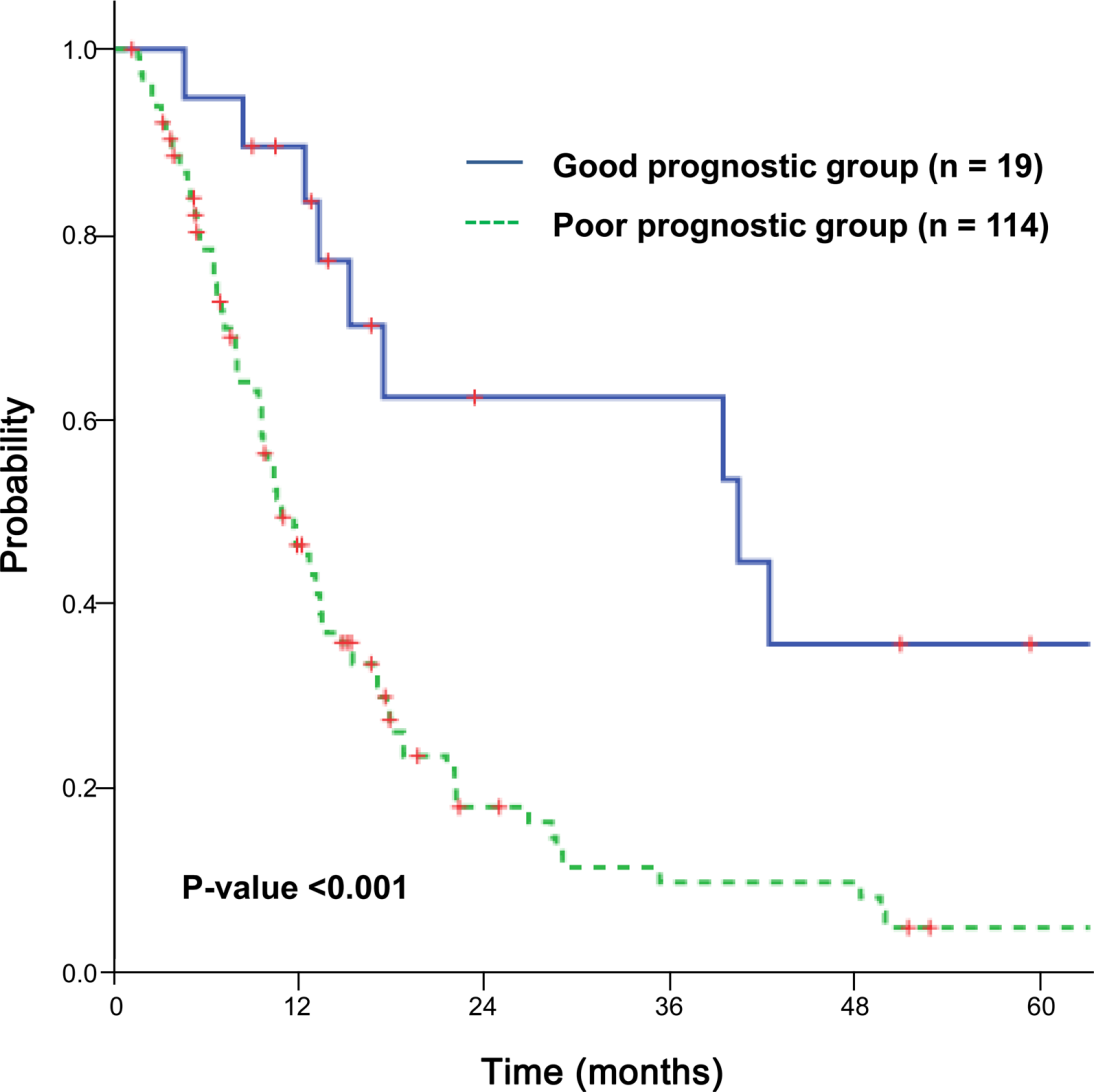
Overall survival curves according to the prognosis. Overall survival curves for the good prognostic group (n = 19) and poor prognostic group (n = 114) (p < 0.001).

We subdivided the patients into a good prognostic group, which was defined as patients with controlled intrahepatic tumors, controlled extrahepatic metastases except for adrenal metastases, and Child-Pugh class A; and the remainder of patients into a poor prognostic group. The median OS of the good prognostic group (n = 19) was significantly longer than that of the poor prognostic group (n = 114) (40.4 vs. 10.8 months, *p* < 0.001) (Fig 2). There was no difference in radiation dose between the two groups. Among the 19 patients of the good prognostic group, there was no correlation between radiation dose and tumor response or OS.

### Adverse effects after radiotherapy

According to CTCAE criteria, acute toxicities, including fatigue, anorexia, nausea, vomiting, and diarrhea, were observed in 90 (67.2%) patients (Table 4). However, most of these adverse events were ≤ grade 2, except for four patients who experienced grade 3 acute toxicities (anorexia in 2, diarrhea in 1, and fatigue in 1). Although grade 3 gastrointestinal bleeding occurred in 4 (3.0%) patients, who received a median dose of 64.9 Gy_10_ (46.8–89.7 Gy_10_), all lesions were controlled with supportive care at each hospital. Therefore, severe adverse events were not observed frequently after radiotherapy for adrenal metastasis.

**Table 4:** Adverse effects after radiotherapy.

Adverse effects		Grade, n (%)			
		1	2	3	4
Acute toxicity	Fatigue	34 (25.4)	10 (7.5)	1 (0.7)	0
	Anorexia	35 (26.1)	23 (17.2)	2 (1.5)	0
	Nausea	36 (26.9)	11 (8.2)	0	0
	Vomiting	12 (9.0)	3 (2.2)	0	0
	Diarrhea	16 (11.9)	2 (1.5)	1 (0.7)	0
Late toxicity	Gastrointestinal bleeding	11 (8.2)	2 (1.5)	4 (3.0)	0

## Discussion

Although the adrenal glands are not an uncommon metastatic site for HCC, it is difficult to establish an optimal treatment strategy for such metastases. In fact, because intrahepatic control of HCC is the most important factor for survival, it is unclear whether variable treatments for adrenal metastasis affect OS or not. There have been limited studies investigating radiotherapy for adrenal metastasis from HCC, however, two studies showed effective symptom palliation after radiotherapy [11,13]. Zeng et al. reported that among 15 patients who suffered from clinical symptoms related to adrenal metastases, including pain in the back/flank, epigastric/upper quadrant visceral pain, or edema of lower extremities caused by the compression of the inferior vena cava, CR occurred in 11 cases who had complete pain relief without medication, good response in two patients, and a complete relief of edema of the lower extremities after radiotherapy [11]. Zhou et al. also demonstrated that all 42 patients with pain in their study experienced pain relief to varying degrees after completion of radiotherapy, even though there was no consistent dose-response relationship [13]. Nevertheless, the actual effectiveness of radiotherapy for adrenal metastasis with respect to tumor response, survival benefit, and prognostic factors, remains unknown.

In this study, the objective response rate was 38.3% according to RECIST criteria, which was quite different from the 68.4–73% reported in previous studies [11,13]. Although the median radiation dose (45 Gy) in this study was lower than in those previous ones (50 Gy), the lower response rate may not result from the lower radiation dose, because there was no significant correlation between response rate and radiation dose. The response rate was also lower than that of radiotherapy for lymph node metastasis from HCC, which had overall response rates of 65.5–96.8% [14–19]. One possible explanation is a discrepancy in imaging follow-up interval or frequency, but this could not be proven. Further study to evaluate any radiobiologic difference in adrenal tissue and to determine dose-response relationship in the context of dose escalation by advanced radiotherapy technique is needed.

We analyzed 134 patients through a multi-institutional study, and found that the median OS was 12.8 months for all patients with adrenal metastasis from HCC. This result was similar to the OS of 10–13.6 months in other studies reporting outcomes of radiotherapy for adrenal metastasis from HCC [11,13]. In the study of Momoi et al., which examined the effectiveness of various treatments for 20 adrenal metastases from HCC, the 1 year OS rates were 51.3% and 42% for adrenalectomy and nonsurgical intervention (TACE, PEI, and radiotherapy), respectively [10]. Park et al. reported that the median OS was 11.1 months among 30 patients with adrenal metastasis; 21.4 months for 5 patients who underwent adrenalectomy; 11.1 months for 19 patients treated with TACE, chemotherapy, and radiotherapy; and 5.7 months for 6 untreated patients [12]. Moreover, retrospective studies on radiotherapy for lymph node metastasis from HCC found similar median survival rates (7–13 months) [14–19]. In the Asia-Pacific sorafenib trial, the median OS of the sorafenib group for lung metastasis was 5.6 months, and that of the sorafenib group for lymph node metastasis was 5.6 months [20]. In addition, Aino et al. reported that the median OS of patients with extrahepatic metastasis, including to lung, bone, lymph node, and/or adrenal gland, was 6.8 months [5]. Taken together, it seems that the survival of radiotherapy for adrenal metastasis from HCC in the present study was not inferior to those of previous studies; however, direct comparisons between studies are not legitimate due to discrepancies among the study cohorts, which included various patient disease statuses and treatment modalities.

Controlled intrahepatic tumor, controlled extrahepatic metastasis, and Child-Pugh class A were independent prognostic factors for OS for patients with adrenal metastasis from HCC in the present study. These prognostic factors corresponded with those of earlier studies on HCC with extrahepatic metastasis including adrenal gland and/or lymph node [4,5,13,14,18]. Zhou et al. demonstrated by multivariate analysis that unfavorable predictors included multiple intrahepatic foci, metastasis to additional organ, and uncontrolled primary HCC [13]. Yoon et al. showed that radiation response and curative treatment aim, defined as well-controlled intrahepatic lesions without extrahepatic metastasis except for lymph node, were significant prognostic factors for survival [14]. Chen et al. reported Child-Pugh class, intrahepatic tumor control, lymph node location, and response to radiotherapy to be prognostic factors for OS in HCC patients who received radiotherapy for lymph node metastasis [18]. Kim et al. demonstrated that Child-Pugh class B and the presence of symptoms were associated with inferior OS after radiotherapy for lymph node metastasis [17]. Prognostic factors for extrahepatic metastasis, especially lymph node metastasis, were still considered as prognosticators in patients with adrenal metastasis. We found that the OS for the good prognostic group was better than that of the poor prognostic group (median OS of 40.4 months vs. 10.8 months).

In this study, most patients achieved local tumor control without progression after radiotherapy, except for 9 patients who suffered disease progression. Among the 19 patients of the good prognostic group with a median OS of 40.4 months, 84.2% achieved disease stability, defined as CR, PR, or SD. Furthermore, only four (3.0%) patients experienced grade 3 acute toxicities, and grade 3 gastrointestinal bleeding occurred in 4 patients, but all lesions were controlled with supportive care. Therefore, severe adverse events were not frequently observed after radiotherapy for adrenal metastasis. Considering these results, radiotherapy for adrenal metastasis from HCC could represent an effective treatment modality with respect to disease stability. Oshiro et al. demonstrated the role of radiotherapy for asymptomatic adrenal metastasis from lung cancer and they concluded that radiotherapy for adrenal metastasis from lung cancer contributed to improved survival, especially for patients with a metachronous metastasis which was shown to be a better prognosis group than those with synchronous metastasis [21]. Therefore, future studies with larger number of patients who have adrenal metastasis from HCC and good prognostic factors are expected to show improved survival.

This study has some limitations. First, the majority of patients had poor prognostic factors, and the response of adrenal metastasis to radiotherapy for such patients is not a critical issue in a real clinical setting. Therefore, regular imaging follow-up, which could reveal objective response, was difficult, and there was insufficient time to evaluate response duration after radiotherapy. Second, as this study included only patients who completed radiotherapy, the prognosis of patients who could not complete radiotherapy due to side effects or other possible causes is unknown. Third, although antiviral therapy is usually used during radiotherapy at most institutions in Korea because there have been many previous reports that radiotherapy is one of the causes of HBV reactivation, the data on antiviral therapy in the study cohort was not evaluated. Lastly, the changes of symptoms from adrenal metastases or biochemical measures of adrenal function were not checked before and after radiotherapy, because we had focused more on the oncologic outcomes, including tumor response, survival rates in the present study. Nevertheless, the current study has several unique strengths. First, the entire study cohort completed radiotherapy; second, tumor responses for all lesions, except one, were evaluated by imaging; and third, a relatively large sample size was achieved through the use of a multi-institutional study.

In summary, although patients with adrenal metastasis from HCC had poor OS, radiotherapy provided an objective response rate of 38.3% and a disease stability of 93.6%, with minimal adverse events. Therefore, radiotherapy for these patients could represent a good treatment modality, especially for those with controlled intrahepatic tumor, controlled extrahepatic metastasis, and good hepatic function.

## Supporting Information

S1 File Raw data excel file for Tables 1–4, Fig 1 and Fig 2. This raw data excel file shows patients characteristics (age, gender, ECOG performance status, viral etiology, status of intrahepatic and extrahepatic diseases, tumor size, location of the tumor, alpha-fetoprotein level, and Child-Pugh class), summaries of the previous of treatments for intrahepatic HCC and radiotherapy for adrenal metastasis, treatment outcomes (response, acute/late toxicities), and survival data for entire patients (n = 134).
(XLSX)
